# Can tetracyclines ensure help in multiple sclerosis immunotherapy?

**Published:** 2021-02-03

**Authors:** Pedro Víctor-Carvalho, Rodolfo Thome, Catarina Rapôso

**Affiliations:** ^1^Department of Structural and Functional Biology, Institute of Biology, University of Campinas, Campinas, SP, Brazil; ^2^Laboratory of Drug Development, Faculty of Pharmaceutical Sciences, University of Campinas, Campinas, SP, Brazil; ^3^Department of Neurology, Thomas Jefferson University, Philadelphia-PA, USA

**Keywords:** Doxycycline, Minocycline, immunomodulation, nonantibiotic actions, neuroprotection

## Abstract

**Background:**

Multiple sclerosis (MS) is a disease of the central nervous system where an autoimmune response leads to chronic inflammation. It represents the second leading cause of non-traumatic disability in the world, affecting mainly young adults and with high female to male incidence. At present, the causative agent in MS is unknown, preventing the development of prophylaxis policies and the understanding of how the human system copes with this complex inflammation. Tetracyclines (Tet) have attracted great attention due to their anti-inflammatory effects. Minocycline and doxycycline represent the second-generation Tet that have been largely used to treat acne and to suppress inflammation. In addition, they are safer and cheaper than other drugs currently used to treat MS.

**Aim:**

This study aims to review recent data involving the Tet minocycline and doxycycline and their therapeutic potential in MS.

**Relevance for Patients:**

Many of the drugs used to treat MS have severe side effects and are costly. Tet, on the other hand, are a safe and inexpensive class of drugs that can modulate the immune response in MS patients.

## 1. Introduction

Besides, the hard historic either in treatment or in diagnosis, new strategies to face multiple sclerosis (MS), have been proposed, with great focus in the repositioning of drugs regularly used for other proposals, such antibiotics. The development from ground zero to a novel drug conducted to treat a specific disease spend huge time and science efforts, leading us to accept and search for more substances to be re-explored. In this review, a collection of evidence to support the use of tetracyclines (Tet) in the MS treatment has been summarized in topics describing the disease, the antibiotics family, and their properties. Furthermore, the study finishes with the last clinical trial results to ensure the knowledge about this approach for Tet.

## 2. MS: An Autoimmune Puzzle

MS is an autoimmune inflammatory disease that directly affects the central nervous system (CNS) through the unregular activity of the immune system [1]. MS typically manifests in sporadic, moderately reversible attacks usually followed by remission. Demyelination, frequently observed in substantia nigra acquired from magnetic resonance imaging (MRI) readings, is normally preceded by inflammation, gliosis, and axonal injury [2]. In the early stages of MS, the demyelination predominates while in advanced stages the axonal loss overlaps. Although the precise mechanisms that lead to MS are unknown and it is believed that loss of blood–brain barrier (BBB) integrity, possibly linked to genetic factors, plays a major role in disease development [3]. Autoreactive CD4^+^ T cells play a major character in MS pathogeny by targeting the myelin sheaths and fueling inflammation in the CNS through the secretion of cytokines and chemokines [3,4]. Glial cells, microglia, and astrocytes are involved in MS pathology through cytokines and growth factors release [5-7]. BBB disruption facilitates the entrance of encephalitogenic T cells and other mononuclear cells into the CNS which contributes to MS pathology [8,9].

MS incidence is higher among young adults and affects women twice than men. MS is the leading cause of non-traumatic neurological disability, and the most common neurodegenerative illness [2,10,11]. According to the World Health Organization [12], MS has become a serious public health problem worldwide, with more than 2.5 million people affected.

The experimental autoimmune encephalomyelitis (EAE) represents the most studied animal model of MS, due to the many similar aspects with the human disease. EAE is induced by subcutaneous immunization of myelin proteins with adjuvants, and many animal species such as mice, rats, and marmoset monkeys are susceptible to this model [13,14].

The pathogenesis of MS is poorly understood but environmental and genetic factors are considered to play an important role in disease development [4]. It is known that latitude may be related to the prevalence of the disease, due to higher latitudes that present lower solar incidence, which means lower Vitamin D production. Thus, a correlation between solar exposure and MS has been established, in which the risk of developing MS is inversely proportional to sun exposure, due to a possible key role of Vitamin D in CNS protection [15-17]. Another factor that would increase not only the pathogenic risk == but the progression, is the smoking habit, especially concerning vascular comorbidities resulting from cigarette consumption [18-20]. Exposure to pathogens, such as Epstein-Barr Virus, has also been associated with an increased risk to develop MS [21]. Genetically, some genes are associated with disease development or worsening, such as the Human Leukocyte Antigen gene, located on the short arm of chromosome 6 (6p21) [22-24].

Therapeutic strategies are considered a major challenge and drugs have been used based on immunological mechanisms [4]. Classically, MS is treated in first-line with Interferon-beta (IFN-β), glatiramer acetate, teriflunomide, and dimethyl fumarate. Second-line therapies include intravenous fingolimod and natalizumab, which have considerable levels of effectiveness and reducing the rate of relapses. Besides, alemtuzumab, cladribine, and ocrelizumab have recently been added as alternative approved therapies. All of these treatments are immunomodulatory or immunosuppressive systemic therapies with high potential for very painful side effects, majorly exhibiting low recovery rates and very expensive coasts to the patients. Unfortunately, only the Relapse Remitting form of MS (RRMS) has these approved therapies [2,25]. Therefore, there is an urgent need for the development of drugs with minimum side effects and that are cheaper enough to ensure more well-being for patients.

Tet are well-known drugs originally used for the treatment of bacterial infections and recent evidence shown that they also possess powerful anti-inflammatory activities. Thus, this study aims to review recent advances and data that demonstrate the anti-inflammatory effects of Tet and its possible use in MS treatment as an adjuvant.

## 3. Tet: Great Pleitropic Antibiotics

As mentioned above, Tet are a group of antibiotics with non-antibiotic properties, such as chemical affinity to numerous proteins and receptors in bacterial and mammalian cells, these characters place Tet to a potential application as MS adjuvant therapy [26]. The first drug of the family was primally discovered on the fermentation products of *Streptomyces aureofaciens*, a soil bacterium, and has also been used for more than a half-century to treat bacterial infections [27,28]. The antibiotic mechanism of action is similar to that presented by aminoglycosides through the binding to the 30S ribosome subunit site where the aminoacyl-tRNA binds, which led to the inhibition of protein synthesis [29]. They are used in the skin, chronic inflammatory airway infections, rheumatoid arthritis, early diffuse scleroderma, and periodontitis treatments [30-32]. Studies in animal models and in *in vitro* approaches suggest viable therapeutic potential in immune-associated diseases, such as diabetes and autoimmune diseases in the nervous system [31,33].

The main non-antibiotic effect is the anti-inflammatory activity that has been shown by many actions in some pathways, highlighting the inhibition of matrix metalloproteinases (MMP) [34] and modulation of cytokines and other pro-inflammatory mediators [35,36]. Furthermore, it has been reported that Tet have pro-apoptotic properties that are very helpful for different approaches, such as for antitumor therapies [37,38].

### 3.1. Pharmacokinetics

**Figure 1:**
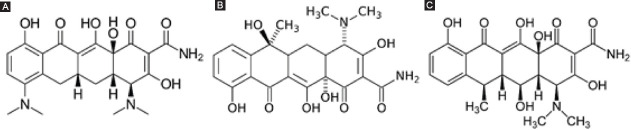
Graphic representation of plane structural formula of drugs from tetracyclines family. A – Tetracycline molecule, B – Minocycline molecule, C – Doxycycline molecule.

Tet are administered orally and have good absorption rates. Among family members, doxycycline (Dox) and minocycline (Min) are almost completely absorbed after ingestion and do not present unexpected reactions when mixed with milk and derivates, in contrast to Tet. Notwithstanding, iron-food consumption is not recommended due to the potential interactions in the Tet molecule that inactivate their abilities (Figure 1) [29,39,40]. The half-life presented by Dox (14-22 h) and Min (11-13 h) is much higher compared to Tet (8.5 h) [29,41]. Their elimination occurs through renal and biliary pathways, with proportions that vary depending on the lipophilicity of the molecule, for example, Dox can be majorly excreted through the intestinal mucosa in the inactive form [42]. Dox and Min as mentioned are lipid-soluble and reach body fluids and tissues with ease and detected at high concentrations in the lymphatic and peritoneal fluids, colonic and prostate tissues, and even in breast milk. Min is the highest lipid-soluble Tet, being 10 times more soluble than Tet itself. Dox has a good lipid solubility as well, 5 times more than Tet, and presents exceptional infiltration in the cerebrospinal fluid, although not greater than Min [29,43-45].

### 3.2. Side effects

Side effects associated with Dox target mainly the gastrointestinal tract when the drug is taken during fasting; however, such discomfort can be bypassed with meal consumption. Likewise, Min ingestion during fasting leads to abdominal discomfort and in high levels, Min can induce vestibular toxicity, which represents the major side effect reported for its oral use [44,46,47]. Moreover, Min intake can lead to adverse reactions that resemble those observed in lupus syndromes, liver dysfunction, and hyperpigmentation, which drives to irreversible color-state change, ranging from grayish to black, manifesting itself in some body parts as well skin, nails, and bones [44,48-50]. Conversely, Dox does not induce these symptoms and is, therefore, safer than Min [44,47].

### 3.3. MMP inhibition

MMPs are a family of 26 proteolytic zinc-binding enzymes, which play important roles in physiological functions such as tissue disruption, reconstruction, and immune responses. MMPs process constituents of the extracellular matrix, remodeling it during either pathological or physiological conditions that include tissue morphogenesis, wound healing, and also cell migration and angiogenesis [51,52]. The conserved pro-domain and catalytic domain are the common characteristics presented among MMPs.

Furthermore, this family is organized into subgroups according to the protein domain and substrate preference, such as gelatinases, stromelysins, collagenases, membrane-type (MT)-MMPs, and the called “other MMPs” [51-53]. After synthesis, most proteins are secreted into the extracellular space by the producing cells, including macrophages, neutrophils, T cells, mast cells, epithelial cells, and mesenchymal cells [54,55].

Many neural cells secrete MMPs during CNS development and its production continues throughout adulthood as the CNS faces challenges and physiological remodeling. However, overexpressed or highly activated MMPs in the CNS are linked to many diseases. Abnormal expression of MMP-3, -7, -9, and -12 is observed in sera from MS patients, and their inhibition alleviates disease severity. Knock-out mice for both MMP-2 (Gelatinase B) and MMP-9 (Gelatinase A) are resistant to EAE while single knock-out mice are susceptible, suggesting that MMP-2 and MMP-9 have a role in inflammation. Notwithstanding, MMP-2 and -9 can induce the expression of chemokines stimulating the PI3K/p-AKT/NF-kb pathway in astrocytes [52,54,56,57].

Moreover, MMPs are involved in BBB disruption by degrading the basement membrane surrounding the endothelium of vessels, thus allowing the entrance of inflammatory cells to the CNS. Inside CNS, high MMP levels worsen the inflammation activating inflammatory mediators. MMPs also disrupt the myelin sheaths contributing to demyelination and neuronal or oligodendrocyte death [54,55,58]. The extracellular MMP inducer (EMMPRIN) modulates activation, proliferation, and invasion of T cells into the CNS contributing to MS pathogenesis. Anti-EMMPRIN treatment reduces EAE severity by downregulating MMP activity. Therefore, as mentioned, MMPs inhibitors can provide beneficial outcomes to MS patients [59].

The use of Tet in this context has been extensively studied and satisfactory results have been reported. In EAE, Min inhibits EMMPRIN, decreases MMP-9 and MMP-2 activities, suppresses the activity of T cells, while also dampening neuronal cell apoptosis [8,60]. In addition, Min upregulates the tissue inhibitor of metalloproteinase 1 (TIMP-1) and TIMP-2 mRNA, potentiating their inhibitory effect on MMPs [8,60].

Inhibitory effects of Dox and Min varies on the differences between MMP species and the pH of the environment. Dox inhibitory effect against collagenases is the highest in all the family. This trend may be explained by a higher affinity of Dox to the ions in the structure of MMPs. Dox promotes the inhibition of MMP-7, MMP-8, and MMP-13 probably through chelation in the structural zinc and/or calcium atoms inserted in the metallic center of the protein, but not in the catalytic zinc site [61]. The pH levels in the microenvironment exert inhibitory effects that can be observed in an experimental assay, in which Dox can inhibit MMP-8 at pH > 7.1, but not in lower pH levels [62]. Curci *et al*. (2000) found an association between a huge reduction in MMP-9 protein and the respective mRNA rates in analyzes after oral Dox administration in patients with abdominal aortic aneurysms. In these patients, Dox decrease monocytic cell levels, as well, inhibited the activation of proMMP-2 in the diseased aortic wall [63]. Studies in human endothelial cells also corroborate the control of MMP-9 expression by Dox [64]. Furthermore, corneal epithelial cell analyses suggest that the MMP inhibition involves blockage in the activation of c-Jun N-terminal kinases (JNK) signaling pathways, which exhibit a key role in the upregulation of MMP [65,66].

### 3.4. Anti-inflammatory effects

#### 3.4.1 Suppression of cytokines and modulation of inflammatory cells

Cytokines consist of almost 300 proteins that play a coordinating role in immune cells, offering complex cascades of events that result generally in a synergistic and balanced action [67,68]. Unbalances in these cytokines, however, can develop exacerbate undesirables and damaging responses [69,70,71]. Although inflammation is necessary to eliminate infectious agents, uncontrolled immune responses lead to autoimmunity and deleterious inflammation. For this reason, inflammation is fine-tuned by signals derived from the environment and cells and, in this case, anti-inflammatory agents are a very important tool to prevent deleterious responses. In MS, autoreactive T cells are stimulated by antigen-presenting cells (APCs), which provide inflammatory cues that trigger T cell differentiation toward an effector helper (Th) profile. These reactive cells secrete the inflammatory cytokines interleukin-17 (IL-17) and IFN-γ that directly impact the integrity of the BBB, in addition to altering the characteristics of CNS resident cells such as astrocytes. Chemokines produced by invading leukocytes and resident stromal cells enhance the influx of lymphocytes and myeloid cells through the BBB, which further perpetuates inflammation. The cells also secrete granulocyte-macrophage colony-stimulating factor (GM-CSF), stimulating a pro-inflammatory profile in monocytes and monocyte-derived cells. These monocytic cells further develop a highly pathogenic behavior, with high production of reactive oxygen species (ROS) and inflammatory cytokines, which, in turn, is related to an enhancement in the inflammation and tissue destruction [71].

The family of Tet has been shown to have significant effects in controlling inflammation by modulating cytokine and chemokine production and nitric oxide levels. Tet also have antioxidant effects. Furthermore, Min has multiple anti-inflammatory properties that include modulation of microglia and immune cells, and reduction in the production of cytokines, chemokines, lipid mediators, and nitric oxide. Pro-inflammatory cytokines, such as TNF-α, IL-1β, and IL-6, are secreted by microglial cells, astrocytes, neutrophils, and macrophages and are closely related in the enhancement of inflammatory responses and overcoming immune reactions. Min suppresses TNF and inducible nitric oxide synthase (iNOS) production and inhibits microglial activation, a key point in the immunopathogenesis of MS [72-74]. Several studies report that Min also decreases the proliferation of T cells [13,75,76]; decreases the expression and production of MHC II, MMP, TNF-α, IL-1β, IL-6, tool-like receptor-2, and iNOS [77-80]; inhibits antigen processing by APCs [81]; decreases the production of MMPs, and protects BBB integrity [13,74,81]; stimulates the induction of Th2 cells at the expense of Th1 cells [74]; provides neuronal and axonal protection by stimulating anti-apoptotic pathways through inhibition of cytochrome c and Smac/DIABLO; as well as inhibiting caspase-1, caspase-3, caspase-8, caspase-9, and decreasing the release of oxygen radicals [65,66,82-87].

#### 3.4.2. Dendritic cells (DC) modulation: a new edge?

DCs are professional APCs present in all tissues of the body. In the presence of microorganisms, DCs trigger innate immune reactions and capture proteins, process antigens and present epitopes in MHC molecules to naïve T-cells, orchestrating adaptive immune responses. DCs are essential in immunity and its regulation. Because of their pro/anti-inflammatory activities, many therapeutic strategies focus their actions on DCs to provide additional modulation on the immune system [88,89]. MS pathogenesis is believed to involve autoreactive T cells that react to myelin proteins and migrate to the CNS to promote damage to the myelin sheaths. In EAE, CD11c^+^ DCs present antigens to T cells and initiate the chronic inflammation observed in the CNS [90]. Bone marrow (BM)-derived DCs treated with chloroquine suppresses EAE by reducing the activation of glial cells, decreasing the gene expression of IL-6 and IL-17, and reducing the infiltration of inflammatory cells in CNS [91]. Similarly, extracts of the murine malaria causative agent, *Plasmodium berghei*, modulated DCs to a tolerogenic profile and, when used in adoptive experimental therapy by transference to EAE mice, stimulates the generation of regulatory T cells (T-reg) and alters the profile of cytokines secreted by T cells promoting disease amelioration [92]. This evidence strengthens the potential of *in vitro* modulated BM-DCs as an efficient cell-based therapy to treat chronic autoimmune diseases. In this context, Min-treated BM-DCs are resistant to maturation stimuli, showing a reduction in MHC II expression and a decrease in cytokines production. Moreover, Min-treated DCs inhibited allogeneic T cell proliferation and induced Treg cells. When injected into EAE mice, Min-treated DCs reduced disease development [93,94]. Combined with Glatiramer Acetate, Min affects the DCs derived from the blood of MS patients, diminishing their ability to present antigens and decreasing their maturation [95].

Dox downregulates CD11c, OX62, CD86, CD80, and MHC II expression on treated DCs. Furthermore, it contributes to an inhibitory profile, which decreases T cell proliferation and the antigen presentation capacity of DCs, constated by low surface costimulatory markers expression [96]. In the presence of Dox, BM-cells were inducible to DCs differentiation and inhibited the RANKL-induced osteoclastic differentiation suppressing MAPKs and c-Fos [97]. As cited before, Dox and Min can decrease the NO amount and the latter plays a key role in the tolerogenic profile in DCs [98], demonstrating another target of Tets.

#### 3.4.3. ROS scavenging action

On the other hand, Dox also decreases iNOS expression, which can contribute to its MMP-inhibition role [63]. Krakauer and Buckley (2003) showed that Dox downregulates IL-1β, IL-6, TNF-α, TNF-γ, MCP-1, MCP-1α, and MIP-1β expression, by interfering with the PKC pathway [99]. In addition, Dox can suppress p38 MAPK and NF-kB pathways, which inhibit the activation of microglial cells, therefore preventing cytokines, chemokines, and many cytotoxic molecules, including NO and ROS [100]. In a model of meningitis, Dox was shown to decrease the levels of IL-1β, IL-6, IL-10, TNF-α, and NO produced by astroglial cells [101]. Dox also decreases caspase-1 expression in humans and the mice systems [78,102]. Due to the relevant immune-suppressive abilities of Dox, it is hypothesized that the drug can be a potential treatment for toxic shock. Furthermore, the intake of lower doses of Dox presents greater anti-inflammatory efficacy than Min, thus having lower toxicity [103].

**Figure 2:**
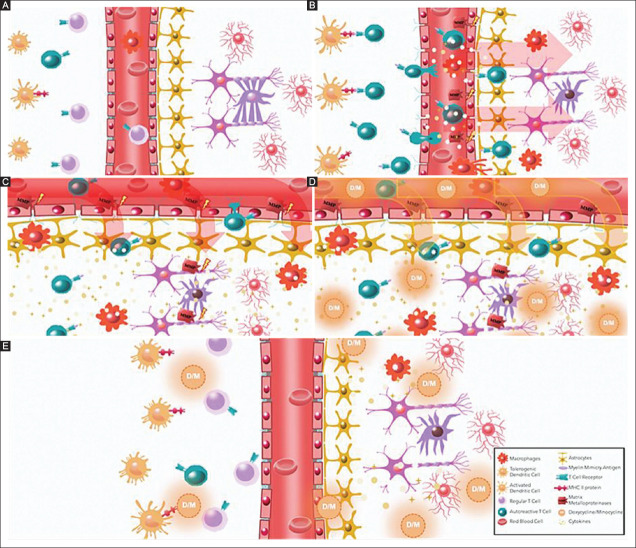
Brain microenvironment in different contexts – A. Regular brain representation showing healthy CNS microenvironment (the right side) and intact occlusion junctions (green lines between endothelium cells, and represented in red) without damage (regular T cells represented in purple) in the BBB; B. Autoreactive T cells (green watercolor cells) activated by an APC cell (e.g., DCs; represented by the orange cells) through an antigen that mimicries myelin protein or another CNS molecule (little purple circumference) trigger inflammatory reactions, enhancement of MMP activity that lies to BBB disruption; also, these T cells can enhance the secretion of pro-inflammatory substances, such as cytokines, chemokines, and nitric oxide (little points in the right side). Furthermore, severe inflammatory reactions can cause injuries over resident cells, including oligodendrocytes and the neuron cell itself; C. Doxycycline and minocycline (orange circles) engage a tolerogenic profile in DCs, which stops the antigen presentation, and also inhibits immune cells, reducing autoreactive T cell proliferation and triggering the stimulation of T naïve profile, inhibition of macrophages (red cells), microglia (pink cells), and astroglia (star-shape yellow cells) cells, MMP (MMP2, MMP3, MMP7, MMP8, MMP9, and MMP13), and cytokines (IL-1β, IL-6, IL-10, TNF-α, TNF-γ, MCP-1, MCP-1α, and MIP-1β); D. After Dox and Min induct a tolerogenic profile, they can keep entering to CNS even after the rebuilding of the BBB, due to their high lipophilicity, protecting patients from relapses.

The probable action mechanisms of Tets are summarized in Figure 2.

### 3.5. Pre-clinical trials

Studies involving Min and animal models show us great possibilities reached by this drug. In the murine EAE model, Niimi, Kohyama, and Matsumoto [8] describe effects in EMMPRIN inhibition, decreased levels in MMP, MMP-2, and T-cell activity, also reduction in neuron cell apoptosis. In this context, Min exerts control in TIMP-1 and -2 expressions, increasing it. In addition, other studies reported the performance in cytokines control (Table 1), in MHC II receptor expression, inhibition in iNOS and caspase pathways, and stimulating the Th2 immune profile, which means a tolerogenic profile [74,78,85]. *In* *vitro* assays, the caspase pathway inhibition and cytokine control are also reported again [77,83,84,87].

**Table 1:** Experimental trials

Tetracycline	Experimental model	Outcome	Reference
Minocycline	EAE	EMMPRIN inhibition; ↓MMP-9 and MMP-2 activity; ↓T cells activity; ↓apoptosis of neural cells;	Niimi, Kohyama and Matsumoto, 2013;
		↑TIMP-1 and TIMP-2 expression	
		MHC II, TNF-α, IL-1β, IL-6, LR-2, and iNOS inhibition	Nikodemova *et al*., 2007; Henry *et al*., 2008;
		↑Th2 immune profile	Popovic *et al*., 2002
		Caspase pathways inhibition	Maier *et al*., 2007;
	*In vitro* assay	Caspase pathways inhibition	Li *et al*., 2002; Scarabelli *et al*., 2004; Wang *et al*., 2004; Wilkins *et al*., 2004;
		iNOS inhibition caspase-1 induction microglial activation	Yrjänheikki *et al*., 1998;
Doxycycline	EAE	↓IL-1β, IL-6, TNF-α, TNF-γ, MCP-1, MCP-1α, and MIP-1β expression	Krakauer and Buckley, 2003
		p38 MAPK and NF- κB pathways suppression	Santa-Cecília *et al*., 2016
		↓IL-1β, IL-6, IL-10, TNF-α, and NO	Muri *et al*., 2019
		↓caspase-1	Fredeking *et al*., 2015
	*In vitro* assay	Caspase pathways inhibition	Gabler *et al*., 1992;
		MMP-7, MMP-8, and MMP-13 inhibition;	
		↓MMP-9 and MMP-2 expression;	Curci *et al*., 2000; Hanemaaijer *et al*., 1998;
		↓iNOS expression	García *et al*., 2005
	Murine forebrain ischemia model	↓caspase-1	Yrjänheikki *et al*., 1998

By the way, Dox treatment in EAE murine model can produce a reduction in cytokine expression, exerting control under its expression, and suppression either in p38 MAPK or NF- kB pathways (Table 2) [101-103,111]. Watching *in vitro* experiments, workgroups reported effects involving control in cytokines expression, MMP inhibition, downregulation in the caspase pathway, and reduction iNOS expression [63,64,86]. In other models such as Murine forebrain ischemia, inhibition of iNOS production is present, additionally the reduction in caspase-1 production [77].

**Table 2:** Clinical trials

Tetracycline	Clinical/Experimental model	Outcome	Reference
Minocycline	Parkinson’s disease	Inhibition of the α-synuclein aggregation;	Schildknecht *et al*., 2011
	Schizophrenia	Improvement in symptoms.	Chaudhry *et al*., 2012
	Acne	↓levels of acne-related lesions	Dreno *et al*., 2001
	Rheumatoid arthritis	Ameliorates the patient conditions	Kaplan *et al*., 1995
	Multiple Sclerosis	↓lesions and risk of relapse; ↓conversion from the clinically isolated syndrome; ↑levels of IL-12p40, which led to the blockage of IL-12p70.	Metz *et al*., 2004;2009; Metz *et al*., 2017; Zabad *et al*., 2007.
Doxycycline	Lyme neuroborreliosis (LNB)	Anti-inflammatory actions; ↓mononuclear cells in CSF;	Bremmel and Dotevall, 2014
	Creutzfeld-Jakob disease	life prolongation in early-stage patients; ↓ evolution in clinical hallmarks; ↓disease worsening.	Varges *et al*., 2017
	Fatal Familial Insomnia	Inhibition of the gene-related expression, as a prophylactic alternative.	Forloni, 2015
	Multiple Sclerosis	↓MMP-9 activity; ↓monocyte migration; inflammatory pathways inhibition; ↓ reduction in lesions.	Minagar *et al*., 2008; Sharafaddinzadeh *et al*., 2010; Silvester, 2005.

EAE: Experimental autoimmune encephalomyelitis; CSF: Colony-stimulating factor

All these results appoint to a great response mediated by the actions of Min and Dox against exacerbating reaction of the immune system, which is essential to stop MS pathogenesis and relapses, as well the development.

### 3.6. Clinical trials

#### 3.6.1 Diseases in general

Clinical trials, the most powerful instrument to ensure the real applicability inside real organisms [104], also had Min and Dox presence. According to ClinicalTrials.gov, there are currently 139 completed clinical trials with Min and the other 29 are in the recruiting phase. Min has been tested on Parkinson’s disease (PD), schizophrenia, acne, cancer, rheumatoid arthritis, autism spectrum disorder, and among other conditions [105]. The NINDS NET-PD Investigators (2006) reports Min and creatine as futile to inhibit the disability progression in PD in both administration times tested, 12 months, and 18 months [107]. A peroxynitrite-removal feature of Min has been reported [106], which can act on the a-synuclein aggregation blocking the nitration in the molecule; which is associated with the cascade reactions that lead to the PD development [108]. In schizophrenia, an improvement in symptoms unaccompanied by detectable cognitive effects was imputed to anti-inflammatory actions of Min [107], however, the administration was rejected by 33% of patients due to side effects proportioned by the Min intake [109]. In a comparative study between Min and zinc gluconate in acne perspective [108], Dreno *et al*. (2001) showed better functional effectiveness of Min in decreasing the number of acne lesions, but with more severe side effects than zinc [110]. In rheumatoid arthritis, Min ameliorates the patient conditions [109,110] and this effect was linked to antibiotic and anti-inflammatory mechanisms [111].

According to ClinicalTrials.gov, there are currently 179 completed clinical trials with Dox that evaluated its effect on polycystic ovarian cancer, Lyme neuroborreliosis (LNB), type II diabetes, aortic aneurysm, periodontitis, coronary artery disease, and Alzheimer’s disease, among others [112]. LNB, a neurological disease developed due to systemic infection by *Borrelia burgdorferi*, involves symptoms such as painful meningoradiculitis and facial nerve palsy. Dox exhibited a great action against the inflammatory condition and reduced the presence of mononuclear cells in the CSF of patients with LNB [113]. In a trial with Creutzfeld-Jakob disease, a transmissible spongiform encephalopathy (TSE) caused by prions, Dox prolonged life in early-stage patients by delaying disease progression [114]. Studying another TSE, Fatal familial insomnia (FFI), also caused by prions, Dox presented actions reporting a possible preventive treatment to patients with a genetically inherited mutation that can lead to FFI development [115]. The sub-antibiotic dose of Dox (SDD) is a no side effect medication with recommended use for chronic inflammatory periodontal disease and chronic inflammatory skin disease, without altering the gut microbiota [116-118]. Dox administration at the SDD level was shown to suppress Graves’ orbitopathy, which is associated with autoimmune Graves’ disease [119].

#### 3.6.2 MS Approach

There are currently four clinical trials evaluating the effect of Min therapy in MS [105]. In these studies, Min promoted a reduction in lesions and decreased relapses with little side effects, prompting its use in MS [120]. Moreover, the same group reported these effects when Min was administered in combination with glatiramer acetate, first-line therapy in MS [121]. Similarly, Min-treated MS patients showed lower lesions sizes and reduced disease severity in the first 6 months of the study, but not in the long-term (24 months) [122]. Importantly, combined with subcutaneous administration of IFN b-1a, Min did not alter MS progression; instead, the patients reported side effects related to the gastrointestinal tract [123]. As a monotherapy, Min decreased frequency of relapses, which was associated with an increase in levels of IL-12p80, which inhibited inflammation, and had little side effects [124].

There is only one clinical trial studying the effect of Dox on MS. According to the study, there is a promising benefit in combining Dox with IFN-β-1a in patients with RRMS, when researchers found that Dox+IFN-β-1a reduced lesion sizes likely by inhibiting MMP-9 activity. Moreover, suppression of monocyte migration through endothelium was reported. Only one patient presented a relapse and insignificant side effects have been noted, additionally, they reported enhancement in IFN-β-1a activity, as well as the reduction in lesions, showed by contrast-enhanced MRI [125]. The combination of Dox and IFN-β-1a blocked inflammation in MS patients by interfering with multiple inflammatory pathways [126].

## 4. Conclusion

MS represents a terrible silent menace that is poorly understood even nowadays; therefore, it is important to develop new therapeutic strategies to complement the current therapies. Tet are a potential allied due to their anti-inflammatory abilities: Cytokines modulation, MMP inhibition, and maintaining the BBB integrity, which prevents immune cells entrance in CNS.

The most understandable collateral effect of Tet misuse is the development of microbial resistance to their antibiotic properties. However, several studies reported that Dox presents valuable clinical effects when administered at very low doses that avoid the antibiotic effect of Tet. Despite that, more studies are required to assess the safest dose and treatment regimen of Dox in MS patients.

Further studies focusing on the inflammatory process, modulation of the immune response, neuroprotective mechanisms, and all actions related to Min and Dox are needed. In this context, the modulation of DCs is especially interesting as a way to circumvent drug toxicity and microbial resistance.

The elucidation of the mechanisms and the comprehension of the behavior of Dox and Min in the long-term administration will provide further evidence to use them in MS therapy. Our research group has an ongoing study with Min and Dox, to evaluate their roles in DCs mechanisms and modulation, avoiding antimicrobial activities, and comparing then, aiming to elect the most secure substance to aggregate in therapeutic strategies. Altogether, we reviewed studies that showed that Tets can represent a cheaper and effective alternative to MS immunotherapy.

### Conflicts of Interests and Acknowledgments

The authors have no conflict of interest regarding the publication of this paper. Furthermore, the authors thank the financial support from the Brazilian National Council for Scientific and Technological Development (CNPq # 131292/2019-6) and The São Paulo Research Foundation (FAPESP #2015/04194-0).
